# AT1 receptor is present in glioma cells; its blockage reduces the growth of rat glioma

**DOI:** 10.1054/bjoc.2001.2102

**Published:** 2001-09-01

**Authors:** E Rivera, O Arrieta, P Guevara, A Duarte-Rojo, J Sotelo

**Affiliations:** Neuroimmunology Unit, National Institute of Neurology and Neurosurgery of Mexico; National University of Mexico, Mexico City, 14269, Mexico

**Keywords:** Losartan, angiotensin, glioma, brain tumour, angiogenesis

## Abstract

Malignancy of neoplasms is partly dependent on angiogenesis. Angiotensin II mediates angiogenesis and transcription of growth-related factors through stimulation of the AT1 receptor (AT1R). Losartan, a drug used mostly for treatment of hypertension, binds strongly to this receptor. We found the presence of AT1 receptor on C6 glioma cells and studied the effect of Losartan on the growth and angiogenesis of C6 rat glioma; Losartan in dose of 80 mg/kg induced 79% reduction of tumoural volume with a significant decrease of vascular density, mitotic index and cell proliferation. Our results demonstrate the conspicuous presence of AT1R in malignant glial cells and a favourable therapeutic response in experimental glioma by selective blockage of the AT1 receptor. © 2001 Cancer Research Campaign  http://www.bjcancer.com


*British Journal of Cancer* (2001) **85**(9), 1396–1399
© 2001 Cancer Research Campaign
doi: 10.1054/ bjoc.2001.2102, available online at http://www.idealibrary.com on IDEAL

http://www.bjcancer.com

### AT1 receptor is present in glioma cells; its blockage reduces the growth of rat glioma

**E Rivera[1], O Arrieta[1,2], P Guevara[1], A Duarte-Rojo[2] and J Sotelo[1,2]**

[1]Neuroimmunology Unit, National Institute of Neurology and Neurosurgery of Mexico and [2]National University of Mexico, 14269 Mexico City, Mexico

**Summary** Malignancy of neoplasms is partly dependent on angiogenesis. Angiotensin II mediates angiogenesis and transcription of growth-related factors through stimulation of the AT1 receptor (AT1R). Losartan, a drug used mostly for treatment of hypertension, binds strongly to this receptor. We found the presence of AT1 receptor on C6 glioma cells and studied the effect of Losartan on the growth and angiogenesis of C6 rat glioma; Losartan in dose of 80 mg/kg induced 79% reduction of tumoural volume with a significant decrease of vascular density, mitotic index and cell proliferation. Our results demonstrate the conspicuous presence of AT1R in malignant glial cells and a favourable therapeutic response in experimental glioma by selective blockage of the AT1 receptor. © 2001 Cancer Research Campaign http://www.bjcancer.com

**Keywords**: Losartan; angiotensin; glioma; brain tumour; angiogenesis

Angiotensin II (AgII) participates on cellular proliferation and angiogenesis (Fernández et al, 1985; Chung et al, 1998; Fournier et al, 1999) through the Angiotensin II receptor 1 (AT1R), whereas the Angiotensin II receptor 2 (AT2R) has a counterregulatory effect (Stoll et al, 1995a, 1995b). The administration of angiotensin converting enzyme inhibitors (ACEI) and AT1R antagonists (AT1RA) block this signal (Burnier, 2001; Stoll et al, 1995a; Wang and Prewitt, 1990; Hafizi et al, 1998). Although the mechanisms involved in the effects mediated by AT1R have not been elucidated it has been found that the stimulation of the receptor increases the transcription of growth-related factors. This mechanism is partly mediated by the platelet-derived growth factor (PDGF) (Wang et al, 1999).

In humans, glioma is the most frequent brain tumour (DeAngelis, 2001). Malignant gliomas show extensive proliferation of vascular endothelium and angiogenesis, essential for tumoral growth and invasiveness. Tumour endothelial cells play an active role in angiogenesis through secretion of growth factors, such as PDGF, vascular endothelial growth factor (VEGF), transforming growth factor (TGFβ), epidermal growth factor (EGF), hepatocyte growth factor/scatter factor (HGF/SF), basic fibroblast growth factor (bFGF), tumour necrosis factor (TNFα) and angiogenin (Jensen, 1998; Schmidt et al, 1999). The interactions of these factors are poorly understood.

A therapeutic possibility in the treatment of cancer is through inhibition of angiogenesis (Arrieta et al, 1998). The present investigation was directed first to search for the AT1R on C6 glioma cells; then, by the use of Losartan ($C_{22}H_{22}CIKN_6O$), a substance with selective affinity for AT1R, we measured its effect on angiogenesis and tumour growth on C6 rat glioma.

*Received 19 February 2001*
*Revised 11 June 2001*
*Accepted 9 August 2001*

*Correspondence to:* O Arrieta
E-mail: ogar@servidor.unam.mx

### MATERIAL AND METHODS

C6 rat glioma cells (American Tissue Culture Collection, Rockville, Maryland, USA) were cultured at 37˚C under sterile conditions in Ham's F-10 medium supplemented with 2.5% fetal calf serum and 15% horse serum. For induction of C6 rat glioma $1 \times 10^7$ cultured C6 cells were inoculated intraperitoneally in a 12-week-old Wistar rat; 20 days later, a large, multilobulated peritoneal tumour developed. The tumour was mechanically dissociated at 4˚C; a suspension of $1 \times 10^7$ cells in 500 μl of saline solution was subcutaneously inoculated into the left thigh of 12-week-old female Wistar rats as previously described (Guevara and Sotelo, 1999). A subcutaneous tumour developed in 80% of the animals, it reached a diameter of 1.5 cm 19–21 days after cell implantation (Guevara and Sotelo, 1999).

Search for AT1R in C6 cells was made by RNA extraction and reverse transcriptase-polymerase chain reactions (RT-PCR). Total RNA was obtained (Chamczynski and Sacchi, 1987) and processed by electrophoresis on formaldehyde-containing agarose gels and visualization with UV light using ethidium bromide staining. RT-PCR were performed with total RNA (Perking Elmer, USA), the primers corresponding to bases 133–150 and 739–719 of the rat AT1 receptor cDNAs (Iwai and Inagami, 1992). The PCR amplification profile consisted of denaturation at 95˚C for 30s, primer annealing at 60˚C for 60s and extension at 72˚C for 90s (28 cycles) using 5 μg total RNA (González-Espinoza and Garcia-Sainz, 1992). The RT-PCR products were electrophorized in 7.5% polyacrylamide gels.

Thirty-seven rats with subcutaneous glioma 1.5 cm diameter were randomly allocated to one of 3 groups: group A controls ($n$ = 12), received orally 0.3 ml of saline daily for 30 days. Group B ($n$ = 13) received 40 mg/kg of Losartan (Merck-Sharp & Dome Pharmaceutics) orally, once a day, for 30 days. Group C ($n$ = 12) received Losartan 80 mg/kg per day at the same schedule. After 30 days of treatment, all animals were anaesthetized with ether and perfused by intracardiac route with 10% formalin in saline solution for histological study. Before perfusion, the animals were bled by intracardiac puncture to study haematological and chemical blood parameters, body weight of animals from all groups remained similar throughout the experiment. The tumour was dissected and its volume determined by water displacement. All, animals used in this study, were handled in accordance to the guidelines of the United Kingdom co-ordinating committee on cancer research (UKCCR) for the welfare of animals in experimental neoplasia (Second Edition) (Workman et al, 1998). For microscopic study the tumour was embedded in paraffin; 10 μm sections were stained with haematoxylin and eosin. Immunohistochemistry with rabbit antibodies to factor VIII R (Harris, 1997) (Sigma), as marker for vascular endothelial cells was used to identify tumour vasculature. Tissue slices of 5 μm were treated with 5% trypsin, washed and incubated with antiserum to factor VIII R for 1 h at 37˚C, followed by incubation with goat antirabbit antibodies conjugated with fluorescein. Sections of rat tongue were used as positive controls. Microvessel density was determined by light microscopy in areas of tumour viability (at the periphery of the tumour) containing the highest numbers of capillaries and small venules (microvessels) per area. These neovascular 'hotspots' were detected by scanning the tumour sections at low power (40 × and 100 ×) and identifying areas having the greatest number of distinct Factor VIII-related antigen-staining microvessels per area. Individual microvessel counts were then made on a 200 × field (× 20 objective and × 10 ocular; equivalent to 0.7386 $mm^2$ per 200 × field) within the neovascular hotspot for each tumour. Any endothelial cell or endothelial cell cluster positive for Factor VIII-related antigen and clearly separate from an adjacent cluster was considered to be a single, countable microvessel. Results were expressed as the highest number of microvessels identified within any single 200 × field (Weidner et al, 1991; Roychowdnury et al, 1996). Cell proliferation was studied by immunohistochemistry with monoclonal antibodies against the proliferation nucleocellular antigen (Sigma, St Louis Missouri, USA). The cell proliferation index was obtained by the mean of positive cells in ten different microscopic fields. All histological evaluations (40 ×) were made without knowledge of the group source of the specimen. Comparisons between groups were made Tukey Test and expressed as means ± standard error (SE); statistical significance was set at 5% level.

### RESULTS

An AT1R fragment of ≈600 bp in C6 glioma cells was detected by RT-PCR (Figure 1, lanes 4 and 7). No such fragments were detected in the absence of reverse transcriptase (Figure 1, lanes 3,5,6). The fragment had the size expected, comparable to that obtained using RNA from rat liver (Iwai and Inagami, 1992).

Administration of Losartan showed a dose dependent reduction in tumoural volume. The mean tumour volume in controls was 51.6 ± 6.4 cc; whereas in animals treated with Losartan 80 mg/kg/d the volume was 11.4 ± 4.4 cc ($P < 0.01$), and in those treated with Losartan 40 mg/kg/d the volume was 31.53 ± 6.9 cc ($P < 0.025$ when compared with controls and $P < 0.01$ when compared with the 80 mg/kg/d group). In rats treated with Losartan at 80 mg/kg/d and 40 mg/kg/d a 79% and 39% reduction of tumour size was obtained, respectively (Figure 2).

In controls the mitotic index in viable areas of tumour was 3.12 ± 0.14; whereas in animals treated with Losartan at doses of 80 mg/kg/d and 40 mg/kg/d it was 1.42 ± 0.12 ($P < 0.01$) and 1.7 ± 0.09 ($P < 0.01$ when compared with controls). The cell proliferation index in tumours from animals treated with Losartan 80 mg/kg/d and 40 mg/kg/d was 39.3 ± 3.5 and 43.8 ± 2.7 respectively ($P < 0.05$); in controls it was 61.2 ± 2.8 ($P < 0.01$ when compared with the experimental groups). The mean number of capillary vessels in tumours from animals treated with Losartan 80 and 40 mg/kg/d was 8.05 ± 0.8 and 10.02 ± 0.5 respectively; in controls it was 14.31 ± 0.81 ($P < 0.001$ when compared with the experimental groups), (Figure 2).

Comparisons of haematological and chemical blood parameters measured at the end of the study showed no differences between groups. No mortality was seen during the experiment.

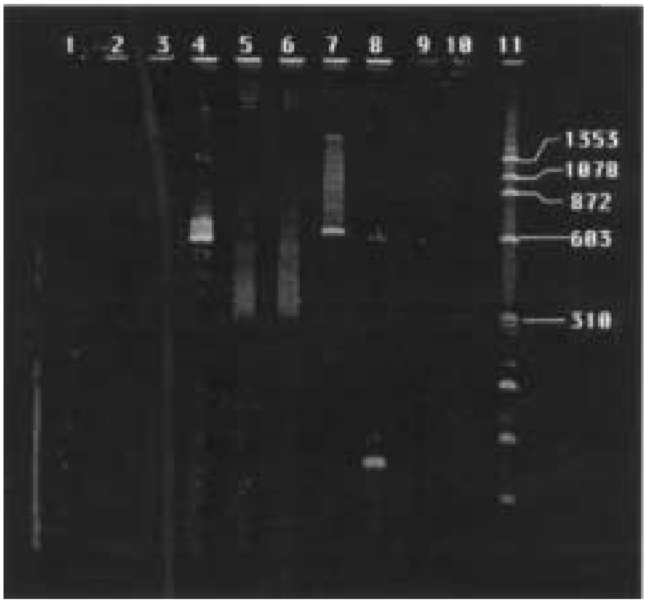

**Figure 1** RT-PCR of the reaction products for the AT1 receptor. Lanes 2 and 6 correspond to the negative control with GADPH in the absence of reverse transcriptase; lanes 8 and 10 correspond to AT1R fragments in the absence of reverse transcriptase, lanes 1,3 and 5 correspond to negative controls with GADPH in the presence of reverse transcriptase; lanes 4 and 7 correspond to AT1R fragments in the presence of reverse transcriptase of ≈600 bp present in C6 glioma cells and lanes 9 and 11 correspond to the negative control and the molecular weight marker with 1353 bp respectively

### DISCUSSION

Our results demonstrated the presence of AT1 receptors in C6 glioma cells; also, the selective blockage of this receptor in experimentally induced C6 glioma reduces angiogenesis and growth. The only clinical precedent to this finding is an apparent low cancer incidence in hypertensive patients receiving ACEI (Lever et al, 1998). Besides its anti-angiogenic activity, Losartan is a blocking agent of cellular signals mediated by AgII; it has a potential antitumoural effect in SH-SY5Y human neuroblastoma cells by inhibition of Angiotensin-converting enzyme and reduction of thymidine incorporation on DNA dependent of AgII (Chen et al, 1991). Our results suggest a similar effect in experimental glioma.

Tumour-induced angiogenesis is a complex phenomenon. In human and experimental glial tumours many cytokines, such as VEGF, PDGF, bFGF, EGF, TNFα and TGF α and β participate in tumour angiogenesis (Lund et al, 1998; Plate et al, 1994a; van der Valk et al, 1997), interestingly, the presence of these cytokines in normal brain is minimal. There is evidence of autocrine and paracrine loops (tumour to tumour, tumour to endothelial cells and endothelial cells to tumour) between these growth factors,

© 2001 Cancer Research Campaign

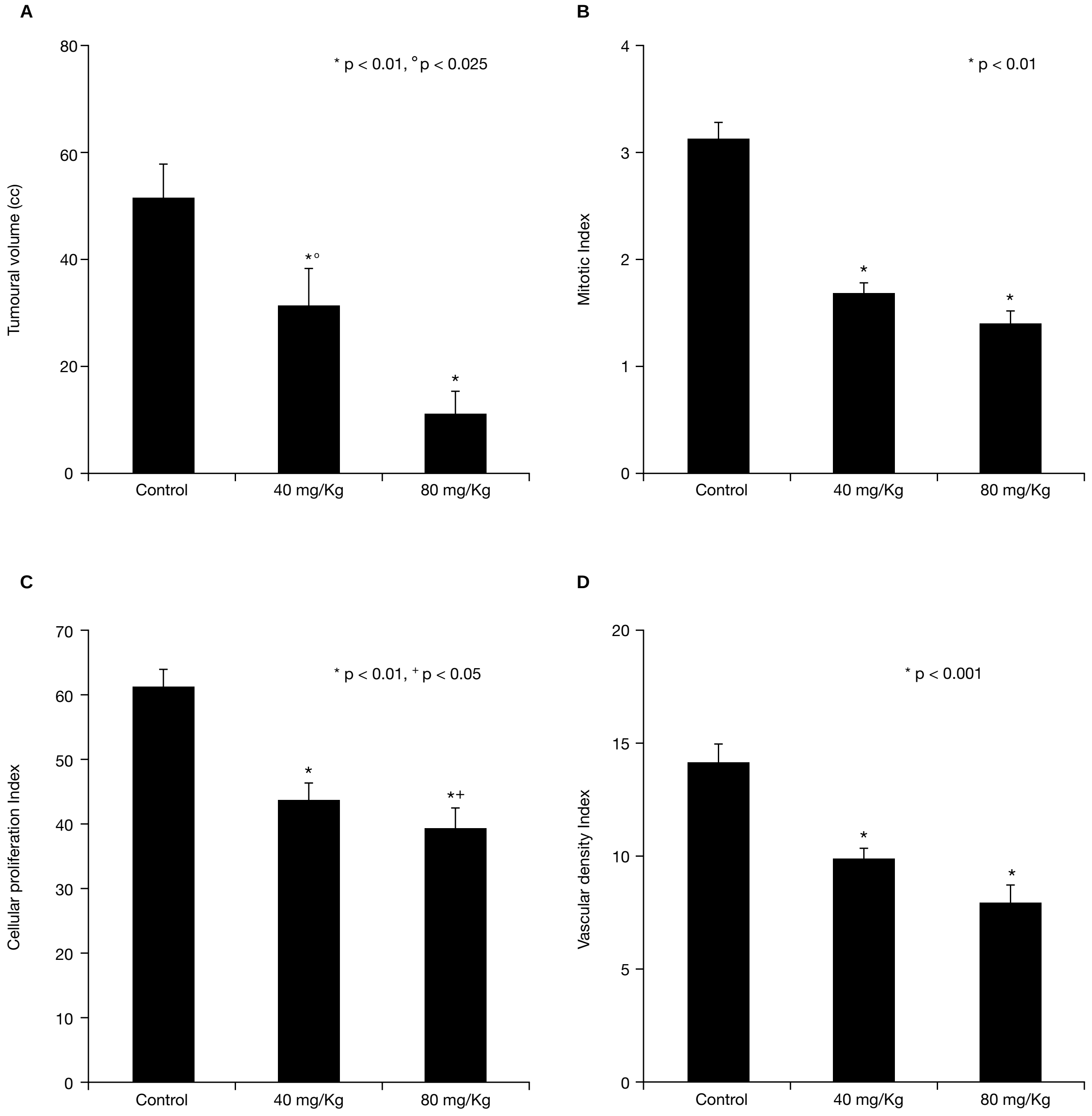

**Figure 2** Comparisons of tumour volume, vascular density, mitotic index and cellular proliferation index in rats with a C6 glioma treated with Losartan at doses of 40 or 80 mg/kg/d and controls particularly PDGF-B/PDGFR (van der Valk et al, 1997), which has been recognized as a growth stimulant for glial cells (Plate et al, 1994a). Aside from promotion of angiogenesis in C6 glioma cells, bFGF, PDGF and heparin binding-EGF increase DNA synthesis. Also, it has been shown that VEGF is highly expressed in glioblastoma multiforme cells, specially around necrotic areas, and flt-1 and flk-1 receptors are abundantly present in endothelial cells from highly malignant gliomas, but not in the normal brain (Plate et al, 1994b). Moreover, VEGF is among the most important mediators of angiogenesis, as shown by the diminution of tumoural growth and angiogenesis after the blockage of this cytokine, either with monoclonal antibodies or with antisense RNA (Kim et al, 1993; Cheng et al, 1996). A similar effect was seen by anti-bFGF antibodies on human glioma cells implanted on nude mice (Lund et al, 1998).

Increase of cell proliferation mediated by AT1 has not been described, although the stimulation of its receptor increases transcription of growth-related factors like *c-fos*, *c-myc* and *c-jun* (Kawakara et al, 1988); and growth-related cytokines like PDGF, bFGF and TGF. In cultured smooth muscle cells treated with AT2 the PDGF-A chain levels increase, as well as the production of PDGF-AA and transforming growth factor 1 (TGF-1) (Dzait et al, 1991). Furthermore, PDGF induces release of VEFG in endothelial cells. This mechanism is activated during periods of angiogenesis on inflammation and glial tumourigenesis (Wang et al, 1999).

© 2001 Cancer Research Campaign

The presence of AT1 receptor on C6 glioma cells may induce the production of PDGF, bFGF, VEGF and TGF1; in turn, it seems likely that the inhibition of this biochemical pathway with Losartan was responsible for the observed decrease of cell proliferation in C6 malignant glioma. Losartan might also induce apoptosis in malignant glial cells by mechanisms similar to those described in other cells (Siragy, 1999). Moreover, the blockade of AT1 induced by Losartan, could also increase the bioavailability of Angiotensin II (by reducing the inhibitory effect on rennin secretion by the AT1R) producing upregulation and an overstimulation of the AT2 receptor, which in turn could potentate the anti-proliferative effects of Losartan. Because the AT1R has also been found in normal astrocytes (Leung et al, 1992) and Losartan penetrates the blood-brain-barrier its effect would be exerted in endothelial cells, as well as in malignant glial cells. A direct effect on glial cells could also be important since those parameters related to mitosis and cell proliferation were significantly reduced (Figure 2).

This is the first report that shows the presence of AT1 in glioma cells, together with the potential anti-tumoural effect of the selective blockage of AT1R disturbing the mechanisms responsible for tumour growth and angiogenesis, which are so conspicuous in malignant glioma. While this manuscript was initially under consideration for publication a report was published showing remarkably similar results about the presence AT1 receptors in pancreatic cancer and inhibition of tumour growth by a receptor antagonist (Fujimoto et al, 2001). It is important to stress the absence of drug toxicity in this study. A clinical trial using Losartan as adjuvant treatment for glioblastoma multiforme and other malignant tumours seems warranted. Additional studies on the effects of Losartan on tumour cells which lack the receptor and/or over express it are necessary. Detection of AT1R in malignant tumours could be a strategy for the use of AT1R blockers as part of treatment.

### ACKNOWLEDGEMENTS

The authors thank Adolfo Gárcia-Sáinz MD, PhD and Daniel Rembao MD for technical advice and histopathological evaluation; and Elena Trueta MD (Merck Sharp & Dome) for providing the Losartan. This work was partly supported by the National Council of Science and Technology of Mexico (CONACyT) L0001–M9608.

### REFERENCES

Arrieta O, Guevara P, Reyes S, Ortiz A, Rembao D and Sotelo J (1998) Protamine inhibits angiogenesis and growth of C6 rat glioma; a synergistic effect when combined with carmustine. *Eur J Cancer* **34**: 2101–2106

Burnier M (2001) Angiotensin II Type 1 Receptor Blockers. *Circulation* **103**: 904–912

Chamczynski P and Sacchi N (1987) Single-step method of RNA isolation by acid guanidinium thyocianate–phenol chloroform extraction. *Anal Biochem* **162**: 156–159

Chen L, Re RN, Prakash O and Mondal D (1991) Angiotensin-converting enzyme inhibition reduces neuroblastoma cell growth rate. *Proc Soc Exp Biol Med* **196**: 280–283

Cheng SY, Huang HJ, Nagane M, Ji XD, Wang D, Shih CC, Arap W, Huang CM and Cavenee WK (1996) Suppression of glioblastoma angiogenicity and tumorigenicity by inhibition of endogenous expression of vascular endothelial growth factor. *Proc Natl Acad Sci USA* **93**: 8502–8507

Chung O, Kuhl H, Stoll M and Unger T (1998) Physiological and pharmacological implications of AT1 versus AT2 receptors. *Kidney Int Suppl* **67**: 595–599

DeAngelis LM (2001) Brain tumors. *N Engl J Med* **2**: 114–123

Dzait VJ, Gibbons GH and Pratt RE (1991) Molecular mechanisms of vascular renin-angiotensin-system in myointimal hyperplasia. *Hypertension* **18 Suppl II**: 100–105

Fernández LA, Twickler J and Mead A (1985) Neovascularization produced by angiotensin II. *J Lab Clin Med* **105**: 141–145

Fournier A, Ghitu A, Darabont R, Mazouz H, Makadassi R, Canaple S, Rosa A and Fernandez LA (1999) Duality of angiotensin II receptors and risk for stroke and cancer: what is the connection? *Presse Med* **28**: 918–922

Fujimoto Y, Sasaki T, Tsuchida A and Chayama K (2001) Angiotensin II type 1 receptor expression in human pancreatic cancer and growth inhibition by angiotensin II type receptor antagonist. *FEBS Lett* **495**: 197–200

González-Espinosa C and García-Sainz JA (1992) Angiotensin II and active phorbol esthers induce proto-oncogenes expression in isolated rat hepatocytes. *Biochim Biophys Acta* **1136**: 309–314

Guevara P and Sotelo J (1999) C6 rat glioma grown into the peritoneal cavity, a large source of tumoral cells for subcutaneous transplant of glioma. *J Neurooncol* **44**: 91–92

Hafizi S, Chester AH, Allen SP, Morgan K and Yacoub MH (1998) Growth response of human coronary smooth muscle cells to angiotensin II and influence of angiotensin AT1 receptor blockade. *Coron Artery Dis* **9**: 167–175

Harris AL (1997) Antiangiogenesis for cancer therapy. *Lancet* **349** (**Suppl 2**): SII 13–15

Iwai N and Inagami T (1992) Identification of two subtypes in the rat type I angiotensin II receptor. *FEBS Lett* **298**: 257–260

Jensen RL (1998) Growth factor-mediated angiogenesis in the malignant progression of glial tumors: a review. *Surg Neurol* **49**: 189–195

Kawakara Y, Sunako M, Tsuda T, Fukuzaki H, Fukumoto Y and Takai Y (1988) Angiotensin II-induced espression of the c-fos gene through protein kinase C activation and calcium ion mobilization in cultured vascular smooth muscle cells. *Biochem Biophys Res Commun* **150**: 52–59

Kim KJ, Li B, Winer J, Armanini M, Gillet N, Phillips HS and Ferrara N (1993) Inhibition of vascular endothelial growth factor-induced angiogenesis suppresses tumour growth in vivo. *Nature* **362**: 841–844

Leung KH, Chang RS, Lotti VJ, Roscoe WA, Smith RD, Timmermans PB and Chiu AT (1992) AT1 receptor mediate the release of prostaglandins in porcine smooth muscle cells and rat astrocytes. *Am J Hypertens* **5**: 648–656

Lever AF, Hole DJ, Gillis CR, McCallum IR, McInnes GT, McKinnon PL, Meredith PA, Murray LS, Reid JL and Robertson JW (1998) Do inhibitors of angiotensin-I-converting enzyme protect against risk of cancer? *Lancet* **352**: 179–184

Lund EL, Spang-Thomsen M, Skovgaard-Poulsen H and Kristjansen PE (1998) Tumor angiogenesis – a new therapeutic target in gliomas. *Acta Neurol Scand* **97**: 52–62

Plate KH, Breier G and Risau W (1994a) Molecular mechanisms of developmental and tumor angiogenesis. *Brain Pathol* **4**: 207–218

Plate KH, Breier G, Weich HA and Risau W (1994b) Vascular endothelial growth factor and glioma angiogenesis: coordinate induction of VEGF receptors, distribution of VEGF protein and possible in vivo regulatory mechanisms. *Int J Cancer* **59**: 520–529

Roychowdhury DF, Tseng Jr A, Fu KK, Weinberg V and Weidner N (1996) New Prognostic Factors in Nasopharyngeal Carcinoma. *Cancer* **77**: 1419–1426

Schmidt NO, Westphal M, Hagel C, Ergun S, Stravrou D, Rosen EM and Lamszus K (1999) Levels of vascular endothelial growth factor, hepatocyte growth factor/scatter factor and basic fibroblast growth factor in human gliomas and their relation to angiogenesis. *Int J Cancer* **84**: 10–18

Siragy H (1999) Angiotensin II receptor blockers: review of the binding characteristics. *Am J Cardiol* **84**: 3S–8S

Stoll M, Meffert S, Stroth U and Unger T (1995a) Growth or antigrowth: angiotensin and the endothelium. *J Hypertens* **13**: 1529–1534

Stoll M, Steckelings UM, Paul M, Bottari SP, Metzger R and Unger T (1995b) The Angiotensin AT2 receptor mediates inhibition of cell proliferation in coronary endothelial cells. *J Clin Invest* **95**: 651–657

van der Valk P, Lindeman J and Kamphorst W (1997) Growth factor profiles of human gliomas. Do non-tumour cells contribute to tumour growth in glioma? *Ann Oncol* **8**: 1023–1029

Wang DH and Prewitt RL (1990) Captopril reduces aortic and microvascular growth in hypertensive and normotensive rats. *Hypertension* **15**: 68–77

Wang D, Huang HJ, Kazlauskas A and Cavenee WK (1999) Induction of vascular endothelial growth factor expression in endothelial cells by platelet-derived growth factor through the activation of phosphatidylinositol 3-kinase. *Cancer Res* **59**: 1464–1472

Weidner N, Semple JP, Welch WR and Folkman J (1991) Tumor Angiogenesis and Metastasis–Correlation in Invasive Breast Carcinoma. *New Eng J Med* **324**: 1–8

Workman P, Twentyman P, Balkwill F, Balmain A, Chaplin D, Double J, Embleton J, Newell D, Raymond R, Stables J, Stephens T and Wallace J (1998) United Kingdom Co-ordinating Committee on Cancer Research (UKCCCR) Guidelines for the Welfare of Animals in Experimental Neoplasia (Second Edition). *Br J Cancer* **77**: 1–10

*© 2001 Cancer Research Campaign* 
